# Response to letter regarding “Validation of a cage‐side agglutination card for *Dal* blood typing in dogs”

**DOI:** 10.1111/jvim.16787

**Published:** 2023-06-15

**Authors:** Marie‐Claude Blais, Emilie Véran

**Affiliations:** ^1^ Univesity of Montreal, Saint Hyacinthe Quebec Canada; ^2^ DMV Center Blainville Quebec Canada

Dear Editors,

Thank you for the opportunity to address Dr Giger's and Dr Freeman's letter.

We appreciate the interest of Dr Giger and Dr Freeman in our publication. We agree with the main point: our publication wrongly refers to the term “validation.” As eloquently explained, a rigorous process is used to establish the validation of a laboratory test, which was not the objective of our study.

The term “evaluation” would have been more appropriate, given that the study goal was to document how the *Dal* typing agglutination card performed in a clinical context. Although the proposal to compare *Dal* typing card performance to the gel technique by standardizing the red blood cell solution hematocrit remains relevant in a laboratory setting, it is not very applicable in clinics because of limited access to a reference laboratory or simply a lack of time when dealing with unstable anemic patients. Thus, our goal was to evaluate the performance of the *Dal* blood typing card in clinically relevant populations of healthy dogs (blood donors or breeds at risk of a *Dal*‐negative phenotype), but also at the cage‐side of the anemic patient.

In this context, our methodology demonstrated that the *Dal* typing card gave very satisfactory results in healthy dogs, but that its interpretation could be problematic in severely anemic dogs (false negative). Simultaneously, a simple dilution protocol using blood samples from healthy dogs allowed us to determine that the PCV threshold allowing reliable interpretation was >20%.

Several specific comments were raised by Drs Giger and Freeman that we will not address specifically, because these points were already raised in the discussion of our article or simply remain semantic details. We leave it to the reader to decide the relative importance of these comments and their impact on our conclusions given that Drs Giger and Freeman come to a similar conclusion: “*If using non‐autoagglutinating (or washed RBCs) blood samples from dogs that are not severely anemic (or adjusted to an appropriate hematocrit), the currently commercially available Dal typing card kit appears to accurately determine the Dal blood type. Therefore, it can be recommended for use in breeds where Dal‐dogs have been recognized*.”

We hope that our publication will promote the use of *Dal* blood typing cards as a tool easily accessible to all clinicians. They remain particularly useful in the context of prevention (i.e., when screening breeds with a high prevalence of *Dal*‐negative individuals and blood donors), given their limitations when used in severely anemic patients and the difficulty in identifying Dal‐negative blood products in emergency situations.

Respectfully submitted,

